# CD44/HA signaling mediates acquired resistance to a PI3Kα inhibitor

**DOI:** 10.1038/s41419-020-03037-0

**Published:** 2020-10-06

**Authors:** Cuixia Yang, Yumeng Sheng, Xiaoxing Shi, Yiwen Liu, Yiqing He, Yan Du, Guoliang Zhang, Feng Gao

**Affiliations:** 1 Department of Molecular Biology Laboratory, Shanghai Jiao Tong University Affiliated Sixth People’s Hospital, 200233 Shanghai, China; 2 Department of Clinical Laboratory, Shanghai Jiao Tong University Affiliated Sixth People’s Hospital, 200233 Shanghai, China; 3 Department of Laboratory Medicine, Shanghai Wujing General Hospital, Shanghai, China

**Keywords:** Breast cancer, Cancer therapeutic resistance

## Abstract

Most luminal breast carcinomas (BrCas) bearing PIK3CA mutations initially respond to phosphoinositide-3-kinase (PI3K)-α inhibitors, but many eventually become resistant. The underlying mechanisms of this resistance remain obscure. In this work, we showed that a CD44^high^ state due to aberrant isoform splicing was acquired from adaptive resistance to a PI3Kα inhibitor (BLY719) in luminal BrCas. Notably, the expression of CD44 was positively correlated with estrogen receptor (ER) activity in PIK3CA-mutant breast cancers, and ER-dependent transcription upon PI3Kα pathway inhibition was in turn mediated by CD44. Furthermore, the interaction of CD44 with the ligand hyaluronan (HA) initiated the Src-ERK signaling cascade, which subsequently maintained AKT and mTOR activity in the presence of a PI3Kα inhibitor. Activation of this pathway was prevented by disruption of the CD44/HA interaction, which in turn restored sensitivity to BLY719. Our results revealed that an ER-CD44-HA signaling circuit that mediates robust compensatory activation of the Src-ERK signaling cascade may contribute to the development of acquired resistance to PI3Kα inhibitors. This study provides new insight into the mechanism of adaptive resistance to PI3Kα inhibition therapy.

## Introduction

Aberrant mutation of PIK3CA (which encodes the p110α subunit of PI3K) is found in 40% of estrogen receptor (ER)-positive breast cancers (BrCas). PI3Kα inhibitors have displayed antitumor efficacy in ER^+^ BrCas with PIK3CA mutation^1,2^. However, limitations including intrinsic or acquired resistance following continuous therapy are emerging^3–5^. Although some progresses have been made^6,7^, the exact mechanisms by which tumor cells escape PI3Kα inhibitors are still unknown.

Recently, cross-talk between the PI3K and ER pathways has been investigated intensively and has provided clues to clinical trials with combinations of inhibitors to PI3K and ER pathways^6^. However, the generation of adaptive resistance to PI3Kα inhibitors with the enhanced ER signaling needs to be addressed. It remains obscure how tumor cells activate ER signaling to induce PI3Kα inhibitors resistance.

CD44, a nonkinase transmembrane receptor that mainly binds extracellular matrix hyaluronan (HA), is preferentially expressed in a variety of tumors, tumor-initiating cells, and drug-resistant tumor lesions^8^. CD44 undergoes extensive alternative splicing during tumor progression, generating two families of isoforms: the CD44 variant (CD44v) and CD44 standard isoform (CD44s). Dysregulation of CD44 alternative splicing between CD44s and CD44v, which is mediated by epithelial splicing regulatory protein 1 (ESRP1), has been reported to be associated with BrCas progression and prognosis^9,10^. More importantly, isoform switching between CD44v and CD44s can trigger the activation of different kinase signaling networks^11^. CD44v was reported to augment mitogen-activated protein kinase (MAPK) signaling and promote cell proliferation^12,13^, while CD44s was believed to stimulate PI3K/AKT activation and render tumor cells insensitive to drug-induced cell death^14^. Because kinase signaling networks are highly dynamic and extremely plastic in response to external stimuli, cancer cells may use their ability to maintain survival signals through the adaptive evolution of kinase circuits upon chemotherapy^15^.

We previously reported that a CD44^high^ state due to alternative splicing could be acquired or lost upon exposure to microenvironmental stimuli^16^. Notably, such switchable phenotypes based on unstable CD44 expression were revealed to give rise to cell plasticity^9^, which might generate adaptive therapeutic resistance and tumor recurrence^17^. In addition, others have reported that interaction of CD44 with HA could promote Src kinase signaling^18^, which in turn affected PI3K/AKT^19,20^ and ERα^21–23^. Therefore, we assume that the inducible acquisition of CD44 and its alternative splicing may play an important role in cancer cell resistance to PI3Kα inhibition.

In this study, we identified a CD44^high^ state that was acquired due to aberrant CD44 splicing upon PI3Kα inhibition. Moreover, crosstalk between CD44 and ER was observed upon PI3Kα inhibition. We demonstrated that CD44-HA could activate a robust compensatory signaling cascade and induce resistance to the PI3Kα inhibitor BYL719. Also, an interconnected feedback loop consisting of CD44-ESRP1-HAS2-ER was found to regulate the transition from a BYL719-sensitive to a BYL719-resistant phenotype. Overall, our study reveals a novel mechanism that links the modification of CD44 splicing patterns with ER signaling.

## Results

### A CD44^high^ state is acquired due to the adaptive resistance to PI3Kα inhibition in luminal breast carcinomas (BrCas)

**Fig. 1:**
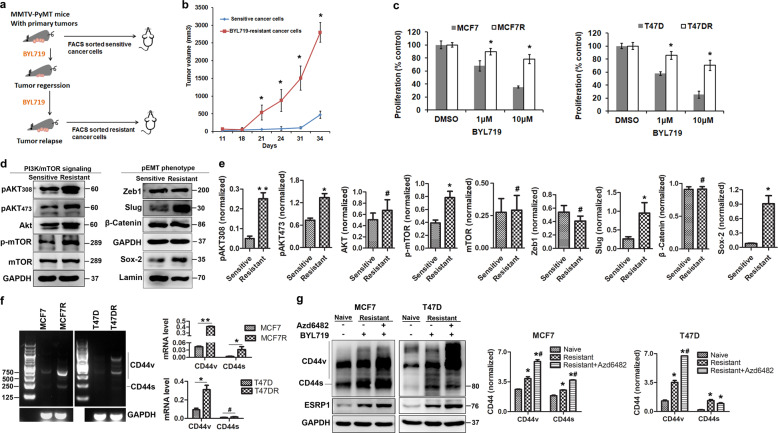
PI3Kα-inhibitory chemotherapy induced an acquired CD44high state and adaptive resistance. **a** Scheme of the in vivo experiment. Schematics of the generation of PI3Kα inhibitor-resistant cells using MMTV-PyMT mouse tumors are shown. Mice were treated daily with BYL719 (25 mg/kg) by oral gavage for 21 days. EpCAM^+^/CD45^−^ cancer cells were sorted from primary MMTV-PyMT tumors or BYL719-resistant tumors and separately orthotopically injected into SCID mice. Tumors were resected and harvested to generate single cells. **b** Tumorigenicity of FACS-sorted BYL719-sensitive or BYL719-resistant cancer cells from MMTV-PyMT tumors following orthotopic injection into SCID mice (*n* ≥ 5/group). Tumor growth was monitored and recorded. Data are represented as the mean (±SD). (statistical analysis: *T*-test, *P < 0.05 vs. control). **c** Proliferation (3 days) of two cell lines sensitive to BYL719 in comparison with their resistant counterparts upon treatment with BYL719 at increasing doses. Each value is the mean (±SD), *n* = 5. (statistical analysis: *T*-test, *P < 0.05 vs. control). **d** Western blot analysis of PI3K/AKT pathway signaling using cell lysates from BYL719-sensitive and BYL719-resistant MCF7 cells and the molecular phenotype as indicated. Levels of the transcription factor Sox-2 and control laminin in cell nuclear extract were determined. **e** Densitometry analysis was performed comparing BYL719-sensitive and BYL719-resistant MCF7 cells based on three biological repeats (statistical analysis: *T*-test, *P < 0.05, **P < 0.01 vs. BYL719-sensitive cells, ^#^*P* > 0.05, not significant). Each value is the mean (±SD) from triplicate samples. **f** The CD44 expression patterns of PI3Kα-resistant luminal breast cancer cells (MCF7 and T47D) were determined by RT-PCR. Results are expressed as mean ± S.D., *n* = 3. *P < 0.05, **P < 0.01, ^#^*P* > 0.05, not significant. **g** Adaptive CD44 alternative splicing upon BYL719 (a p110α inhibitor) treatment or combined treatment with BYL719 and Azd6482 (a p110β inhibitor) in BYL719-resistant cells (MCF7R and T47DR) was assessed by Western blot analysis. Results are expressed as mean ± S.D., *n* = 3. *P < 0.05 when compared with naive MCF7 cells. ^#^*P* < 0.05 when compared with BYL719-resistant cells without Azd6482 treatment.

To address resistance to BYL719, we orthotopically transplanted purified BYL719-sensitive and BYL719-resistant luminal cancer cells from MMTV-PyMT tumors into severe combined immunodeficient (SCID) mice (Fig. 1a). Our data indicated that the BYL719-resistant cells exhibited a stronger tumor-forming ability (Fig. 1b). Then, luminal-like BrCa cells bearing mutated PIK3CA (MCF7 and T47D cells) were selected and subjected to treatment with BYL719 at increasing concentrations over time until resistance occurred. Our results showed that the resistant cells failed to undergo growth arrest in response to BYL719 exposure (Fig. 1c).

Next, cell growth signaling and phenotype of BYL719-resistant cells were analyzed. Results showed that AKT and mTOR were significantly activated in the resistant cells (Fig. 1d). To analyze the molecular phenotypes, we assessed expression of the mesenchymal markers. Our results showed that Slug and SOX-2 were significantly increased, while no significant changes were observed in Zeb1 and β-Catenin (Fig. 1d and e). Therefore, these resistant cells displayed a hybrid epithelial/mesenchymal (E/M) state.

To further elucidate the nature of the adaptive resistance, we determined the changes in CD44 expression patterns. We found that the expression of both CD44s and CD44v was significantly increased in the resistant cells (Fig. 1f and g), indicating that aberrant alternative splicing generated a resistance-specific CD44^high^ state. Interestingly, the splicing factor ESRP1 was also significantly upregulated. The parallel increases in CD44s and ESRP1 further supported the notion of CD44 abnormal splicing.

We next determine whether the CD44^high^ state was caused by rebound activation of the p110β isoform upon BYL719 treatment^24^. Combination of BYL719 and the PI3Kβ inhibitor AZD6482 further upregulated the expression of CD44 (Fig. 1g), suggesting that the increased CD44 alternative splicing was directly related to PI3K inhibition. Therefore, the CD44^high^ state might play a role in mediating resistance to PI3Kα inhibition.

### CD44 is positively correlated with ER in PIK3CA-mutated breast cancers

**Fig. 2:**
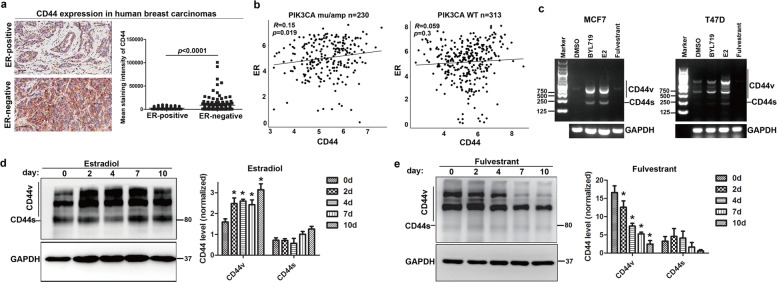
The expression of CD44 was positively correlated with ER activity. **a** CD44 expression in human breast tumor sections based on ER+ (*n* = 129) or ER− (*n* = 112) cells was determined by immunohistochemistry. The intensity of CD44 expression was measured by integral optical density^46^ using Image-Pro Plus software. Data were analyzed with the Mann-Whitney test. Scale bars, 25 µm. **b** Correlations between CD44 and ER expression in PIK3CA-mutant and PIK3CA-WT breast cancers (TCGA) are shown. The Spearman correlation coefficient (R) and corresponding p-value for the mRNA expression of CD44 with ER were analyzed from RNA sequencing data from primary breast tumors in TCGA. **c** ER regulated the alternative splicing of CD44. The expression of CD44 variants and ESRP1 in MCF7 and T47D cells depleted of hormones for 3 days and treated with DMSO, E2 (100 nm), BYL719 (1 μM), or fulvestrant for 4 days was measured by RT-qPCR. Data are representative of at least three biological repeats and results are expressed as mean (±SD), *n* = 3. *P < 0.05. **d**, **e** The expression patterns of CD44 in response to estradiol (E2) or fulvestrant were observed by immunoblotting in MCF7 and T47D cells treated with E2 or fulvestrant for 0, 2, 4, 7, or 10 days. The CD44 bands were quantified by densitometry analysis and normalized against GAPDH. Three independent experiments were used to obtain the average. The means (±SD) are plotted; *P < 0.05 vs. control.

As enhanced ER activity was revealed to contribute to resistance to PI3Kα inhibition^6,25^, we then explored the relationship of the simultaneous adaptive upregulation of CD44 and ER upon BYL719 treatment. CD44 expression in ER^+^ (*n* = 129) or ER^−^ (*n* = 112) tumor samples was assessed by immunohistochemical analysis. The results showed that the presence of CD44^high^ cells in the ER^+^ tumors was significantly lower than that in the ER^−^ BrCas (Fig. 2a and Supplementary Table 1). Intriguingly, additional analysis focused on luminal-like BrCas from The Cancer Genome Atlas (TCGA) database indicated a significant positive correlation between the expression of CD44 and ER in patients bearing either PIK3CA mutation or amplification, whereas no significant correlation was found in patients without PIK3CA mutations (Fig. 2b).

Subsequently, we studied the effects of ER activation on CD44 splicing. The results revealed the significant estradiol-mediated and BYL719-mediated increase of CD44 alternative splicing. Meanwhile, fulvestrant markedly suppressed CD44 isoform splicing (Fig. 2c and e). These results demonstrated that ER activation is of great importance in regulating CD44 alternative splicing.

### CD44 mediates ER-dependent transcription upon PI3Kα inhibition

**Fig. 3:**
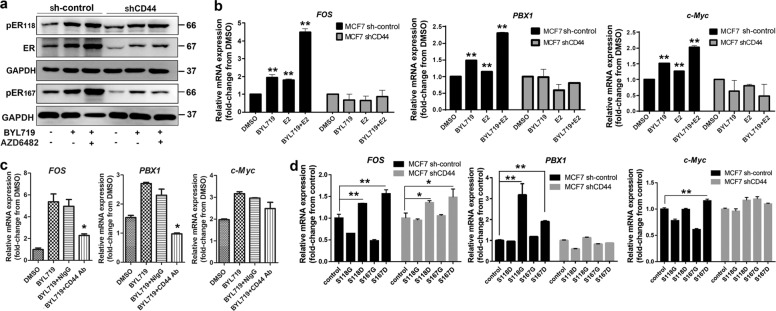
CD44 mediates ER-dependent transcription upon PI3Kα pathway inhibition. **a** Knockdown of CD44 expression attenuated ER activation triggered by PI3K inhibitors. Expression of ER and phosphorylated ER (Ser118 and Ser167) in control or sh-CD44-transfected MCF7 cells depleted of hormones for 3 days and subsequently treated with DMSO, BYL719 (1 μM), or combination BYL719 and Azd6482 (1 μM) treatment for 3 days was measured by immunoblotting. **b** Expression of candidate target genes in control or sh-CD44-transfected MCF7 cells depleted of hormones for 3 days treated with DMSO, E2 (100 nM), BYL719 (1 μM), or BYL719 combined with BYL719 (1 μM) and E2 (100 nM) for 24 h was measured by RT-qPCR. Values are presented as the mean (±SD). The *P*-values were calculated using Student’s *t*-test. All data represent at least three biological repeats. *P < 0.05, *P < 0.01 vs. control. **c** Expression of candidate target genes in cells depleted of hormones for 3 days and treated with DMSO, BYL719 (1 μM), anti-CD44 antibody (25 μg/ml), or both BYL719 (1 μM) and CD44 antibody (25 μg/ml) for 24 h was measured by RT-qPCR. Normal mouse IgG (NIgG) was used as negative control. Values are presented as the mean (±SD). Results are expressed as mean (±SD), *n* = 3. *P < 0.05 when compared to BYL719 treatment with or without NIgG. **d** Effects of phosphomimetic and phospho-dead ER on the transcription of ER target genes in naïve MCF7 or sh-CD44 MCF7 cells. Exogenously phosphomimetic and phospho-dead ER (S118 and ER S167) were prepared. Two codons of ESR1 gene encoding for serine (S) residues were mutated to encode either an aspartic acid (D) residue (ER118S→D, ER167S→D) or glycine (G) residue (ER118S→G, ER167S→G). The phosphomimetic (S118D and S167D) and phospho-dead (S118G and S167G) were expressed in cells by lentivirus transfection. The expression of ER target genes was then measured by RT-qPCR. An empty vector was used as a negative control (vector). Values are presented as the mean (±SD). All data represent at least three biological repeats. *P < 0.05, *P < 0.01.

To further confirm the correlation between CD44 and ER, CD44 was knocked down by lentivirus transfection. CD44 knockdown resulted in a reduction in ER expression (Fig. 3a). Phosphorylation of ERα at the major N-terminal domain sites Ser104, Ser106, Ser118, and Ser167 was then analyzed. The results indicated that Ser104 or Ser106 phosphorylation of ERα was not associated with PI3K inhibition or CD44 knockdown (Supplementary Fig. 1a). Notably, the increase in ERα expression and phosphorylation (Ser118 and Ser167) upon BYL719 treatment was attenuated in CD44-knockdown cells (Fig. 3a). To further elucidate this effect of CD44 on ER activity, ER-dependent transcription was determined in the presence of estradiol, BYL719, or both. As expected, increases in the candidate target genes PBX1, cFOS, and c-MYC upon estradiol or BYL719 stimulation were impaired after CD44 silence (Fig. 3b). Also, similar effects on cell cycle were noted in CD44-knockdown cells treated with estradiol or BYL719 (Supplementary Fig. 1b).

Furthermore, an antibody blocking CD44 dramatically inhibited the upregulation of ER target genes upon BYL719 treatment (Fig. 3c). Given that pER (Ser118) and pER (Ser167) levels were increased by BYL719 treatment in CD44-knockdown cells, we further investigated the role of pER on CD44/ER-dependent transcription by overexpressing phospho-dead and phosphomimetic mutants (Supplementary Fig. 2). Our results showed that CD44 knockdown attenuated phosphomimetic ER (Ser118D and Ser167D)-induced transcriptional activation of ER targets (cFOS, PBX1, and c-MYC) (Fig. 3d). As expected, phospho-dead ER (Ser118G and Ser167G) could not induce the transcription of ER target genes in naïve or CD44-knockdown cells. These results suggested that the enhanced ER-dependent transcription upon PI3Kα inhibition was attenuated by CD44 knockdown.

### CD44 aberrant splicing is sufficient to limit the sensitivity of luminal BrCa cells to BYL719

**Fig. 4:**
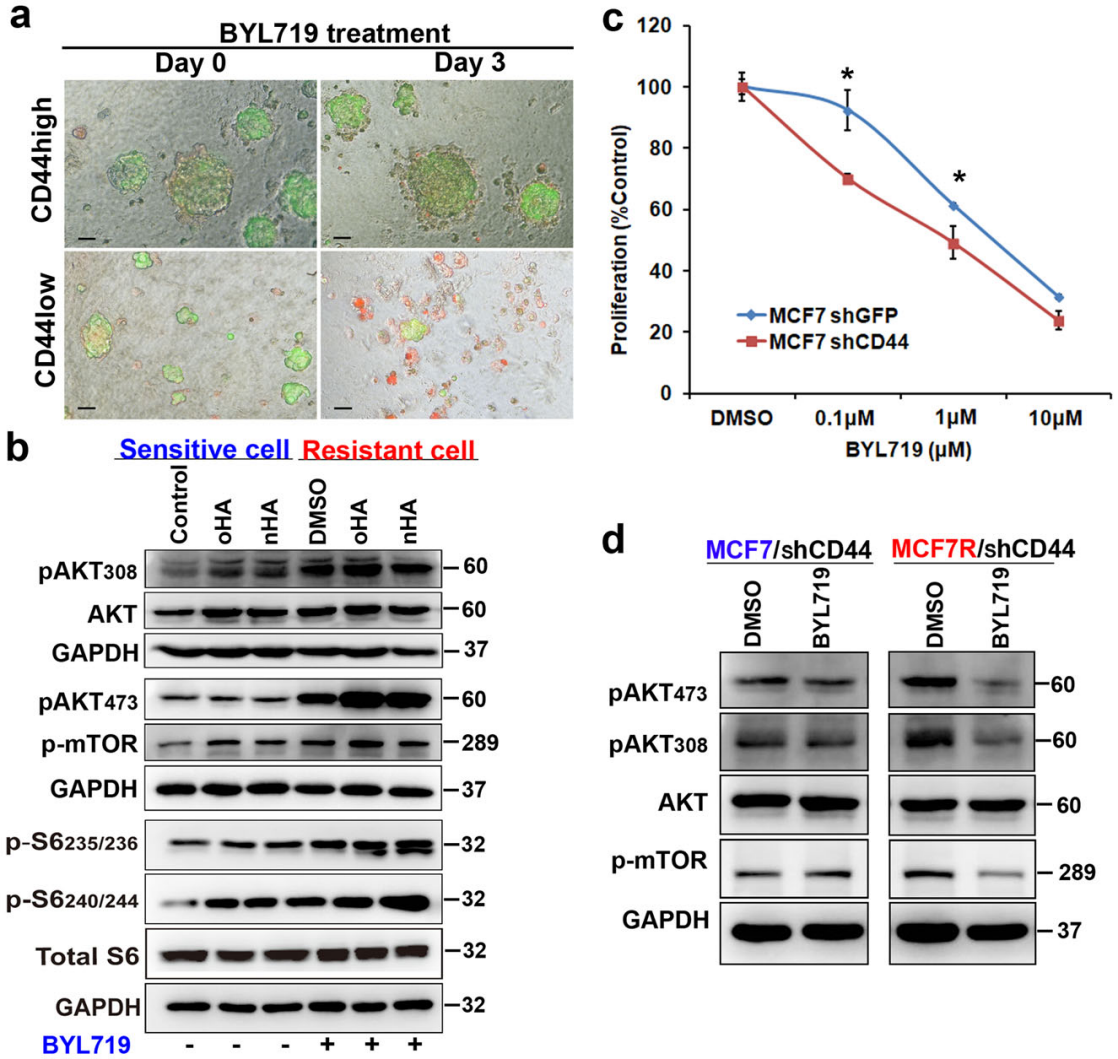
CD44 activation was sufficient to limit the sensitivity of luminal breast cancer cells to BYL719. **a** Cytotoxicity of FACS-sorted EpCAM+/CD45−/CD44^high^ or EpCAM+/CD45−/CD44^low^ cancer cells from MMTV-PyMT mice upon treatment with 1 μM BYL719 was determined in a 3D culture system using an in situ cell death detection kit on the indicated days. Scale bars, 50 µm. **b** AKT/mTOR signaling was evaluated in naïve or BYL719-resistant MCF7 cells treated for 4 h with 1 μM BYL719 in the presence of 10 μg/ml oHA or 10 μg/ml HA as indicated. **c** Dose-response curves from MCF7 cells transfected with shGFP and sh-CD44 and treated with BYL719 for 4 days. Data are represented as the mean (±SD). *p < 0.05 vs. control, *n* = 5. **d** Western blot to compare MCF7 and MCF7R cells transfected with sh-CD44 and treated with BYL719 (1 μM) for 3 days.

To determine the relationship between BYL719-resistance and the acquired CD44^high^ state, either CD44^high^ or CD44^low^ subpopulations of primary tumor cells were purified^16^ and cultured in a low-attachment plate to form cell spheroids. Our results showed that spheroids from the CD44^high^ subset grew and progressed more rapidly on collagen/Matrigel 3D matrix than those from the CD44^low^ subset (Fig. 4a). Besides, the CD44^high^ subset was more resistant and less responsive to BYL719 than the CD44^low^ subset (Fig. 4a), suggesting that the acquired CD44^high^ state is related to BYL719-resistance.

To further understand the role of CD44 in adaptive resistance, HA at a range of different molecular weights (high-molecular-weight HA and low-molecular-weight HA), which closely mimics the tumor microenvironment, was added to activate CD44 signaling pathways. As indicated in Fig. 4b, HA treatment significantly activated PI3K/AKT/mTOR signaling in naïve MCF7 cells. Moreover, this activation of CD44 either partially or completely attenuated the BYL719-induced inhibition of AKT signaling (Supplementary Fig. 3), suggesting that CD44/HA signaling contributes to generating adaptive BYL719-resistance. Notably, in BYL719-resistant cells, HA further enhanced the rebound activation of PI3K/AKT/mTOR signaling (Fig. 4b). Furthermore, CD44 knockdown was sufficient to decrease cell viability in response to BYL719 treatment (Fig. 4c), and additional treatment with HA was incapable of interfering with BYL719-induced inhibition of AKT/mTOR (Supplementary Fig. 4). The combination of CD44 knockdown and BYL719 treatment decreased the phosphorylation of AKT and mTOR in resistant cells (Fig. 4d). Taken together, a CD44^high^ state in adaptive resistant cells might mediate BYL719 resistance.

### Adaptive CD44^high^ expression in response to PI3K inhibition is regulated by HA/HAS2 in a positive feedback loop

**Fig. 5:**
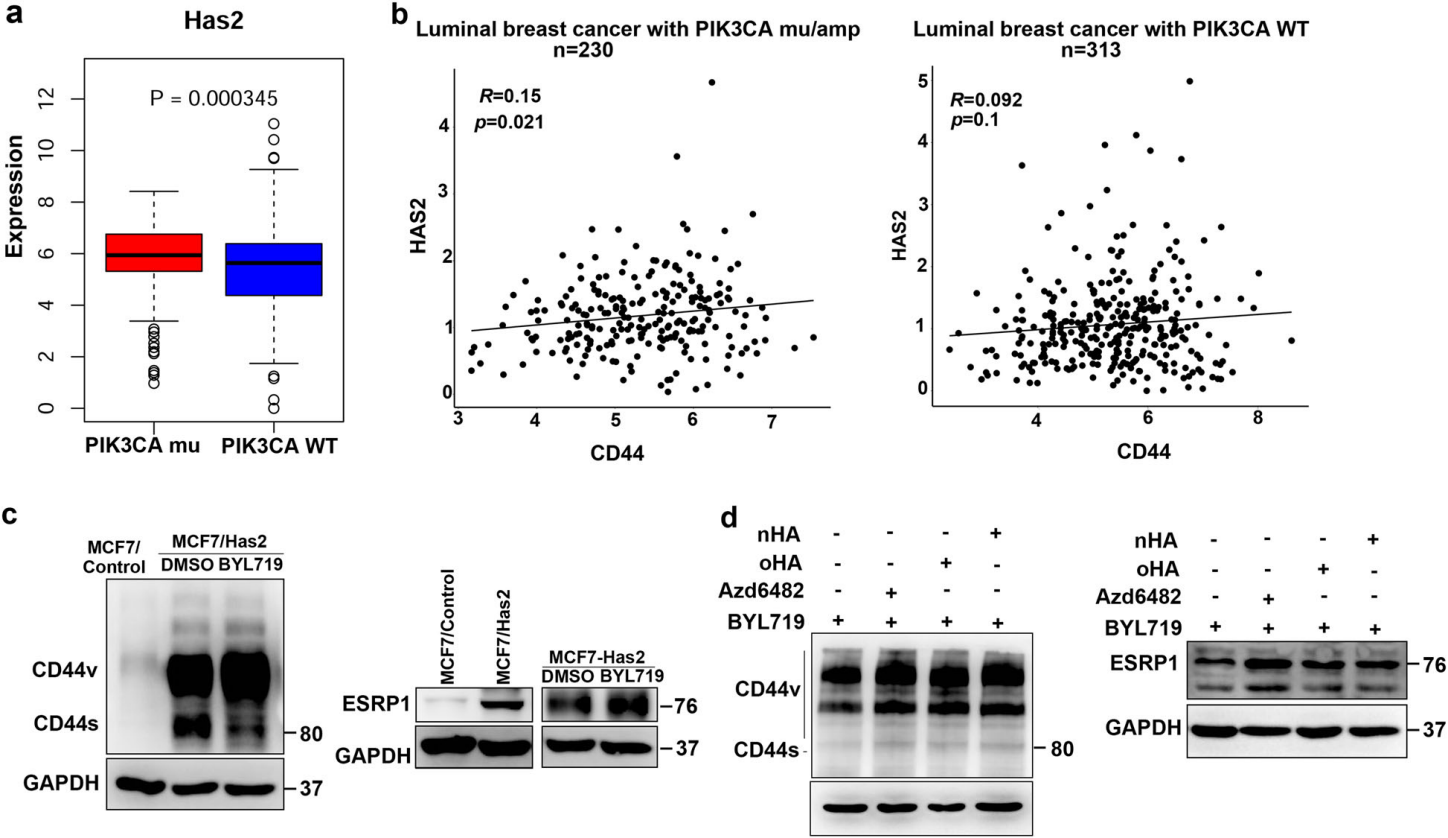
Adaptive CD44^high^ expression in response to PI3K inhibition is regulated by HA/HAS2 in a positive feedback loop. **a** Upregulation of HAS2 in luminal-like breast cancers with mutant PIK3CA from TCGA data. **b** Correlations between HAS2 and CD44 expression in PIK3CA-mutant and WT PIK3CA breast cancers (TCGA) are shown. The Spearman correlation coefficient (R) and corresponding p-value for the mRNA expression of HAS2 with CD44 were analyzed from RNA sequencing data from primary luminal-like breast tumors in TCGA. **c** The expression of CD44 and ESRP1 were determined in MCF7/HAS2 cells treated with BYL719. **d** The expression of CD44 and ESRP1 upon BYL719 (1.0 μM) treatment or combination treatment with the p110β inhibitor Azd6482 (1.0 μM), oHA (10 μg/ml), or HA (10 μg/ml) in BYL719-resistant T47D cells were determined by immunoblotting.

To further investigate the mechanisms leading to CD44 activation during the acquisition of resistance to PI3Kα inhibition, we focused on whether an HA-CD44 axis contributes to the adaptive resistance. HA synthase 2 (HAS2) acts as the main source of HA. Analysis of TCGA data revealed that HAS2 was more highly expressed in luminal BrCas with PIK3CA mutations than in those without PIK3CA mutations (Fig. 5a). Consistently, the expression of HAS2 was highly increased in BYL719-resistant cells (Supplementary Fig. 5), implying an increase in secreted HA. More importantly, high HAS2 expression was positively associated with the expression of CD44 in luminal BrCas with either PIK3CA mutation or overexpression (Fig. 5b).

We next attempted to determine whether HAS2 plays a role in modulating CD44 splicing upon PI3Kα inhibition. Results showed that cells with stable HAS2 overexpression presented high expressions of CD44 and ESRP1 (Fig. 5c). Interestingly, subsequent treatment with BYL719 led to further increases in CD44 and ESRP1 expression (Fig. 5c). Furthermore, in BYL719-resistant cells, cotreatment with exogenous HA and BYL719 also resulted in further increases in CD44 and ESRP1 (Fig. 5d), which is identical to the effects of combination treatment with BYL719 and the PI3Kβ-specific inhibitor Azd6482 (Fig. 5d). These results implied that upon BYL719 treatment, HAS2/HA upregulates the splicing of CD44 in a positive feedback loop.

### The enhanced CD44-HA interaction activates AKT/mTOR and attenuates sensitivity to BYL719

**Fig. 6:**
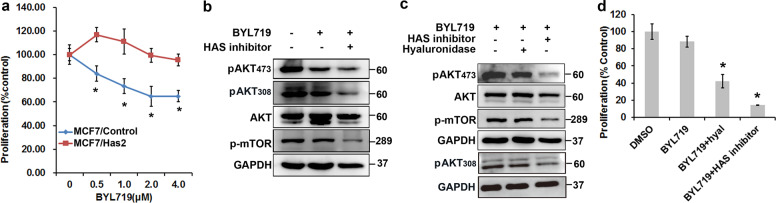
The enhanced CD44-HA interaction activates AKT/mTOR and attenuates sensitivity to BYL719. **a** Growth curves of MCF7 cells stably expressing HA synthase 2 (HAS2) or naïve MCF7 cells treated with BYL719 at increasing concentrations for 4 days. Data are represented as the mean (±SD). *p < 0.05. **b** AKT/mTOR signaling was evaluated in MCF7/HAS2 cells treated with 1 μM BYL719 with or without an HAS inhibitor (4-methylumbelliferone, 0.2 μM) for 3 days. **c** AKT/mTOR signaling was evaluated in MCF7/HAS2 cells treated with BYL719 (1 μM), BYL719 (1 μM) plus hyaluronidase (hyal), or the combination of BYL719 and an HAS inhibitor (0.2 μM) for 24 h. **d** The proliferation of MCF7/HAS2 cells treated with BYL719 (1 μM), BYL719 (1 μM) plus hyaluronidase (hyal), or the combination of BYL719 and an HAS inhibitor (0.2 μM) for 3 days was determined. Each value is the mean (±SD). *p < 0.05 vs. control, *n* = 5.

We next explored the effect of the enhanced HA-CD44 interaction on cell sensitivity to BYL719. Our results showed that forced stable expression of HAS2 was sufficient to limit sensitivity to BYL719 (Fig. 6a) and reduced BYL719-mediated inhibition of AKT and mTOR (Fig. 6b). Furthermore, when HA expression was subsequently degraded or inhibited by hyaluronidase or HAS inhibitor, the attenuated inhibition of BYL719 was rescued (Fig. 6c), and the change in resistance was reversed (Fig. 6c and d). Interestingly, hyaluronidase treatment only slightly inhibited AKT/mTOR activation in MCF7/HAS2 cells upon BYL719 treatment (Fig. 6c), although it obviously inhibited proliferation (Fig. 6d). This discordance might be due to relatively insufficient of exogenous hyaluronidase comparing to overexpressed HAS2 for sustaining AKT/mTOR inhibition over the exposure period. Taken together, these results suggested that enhanced CD44/HA signaling leads to sustained AKT and mTOR activation.

### CD44/HA binding activates the Src-ERK-Ezrin signaling cascade

**Fig. 7:**
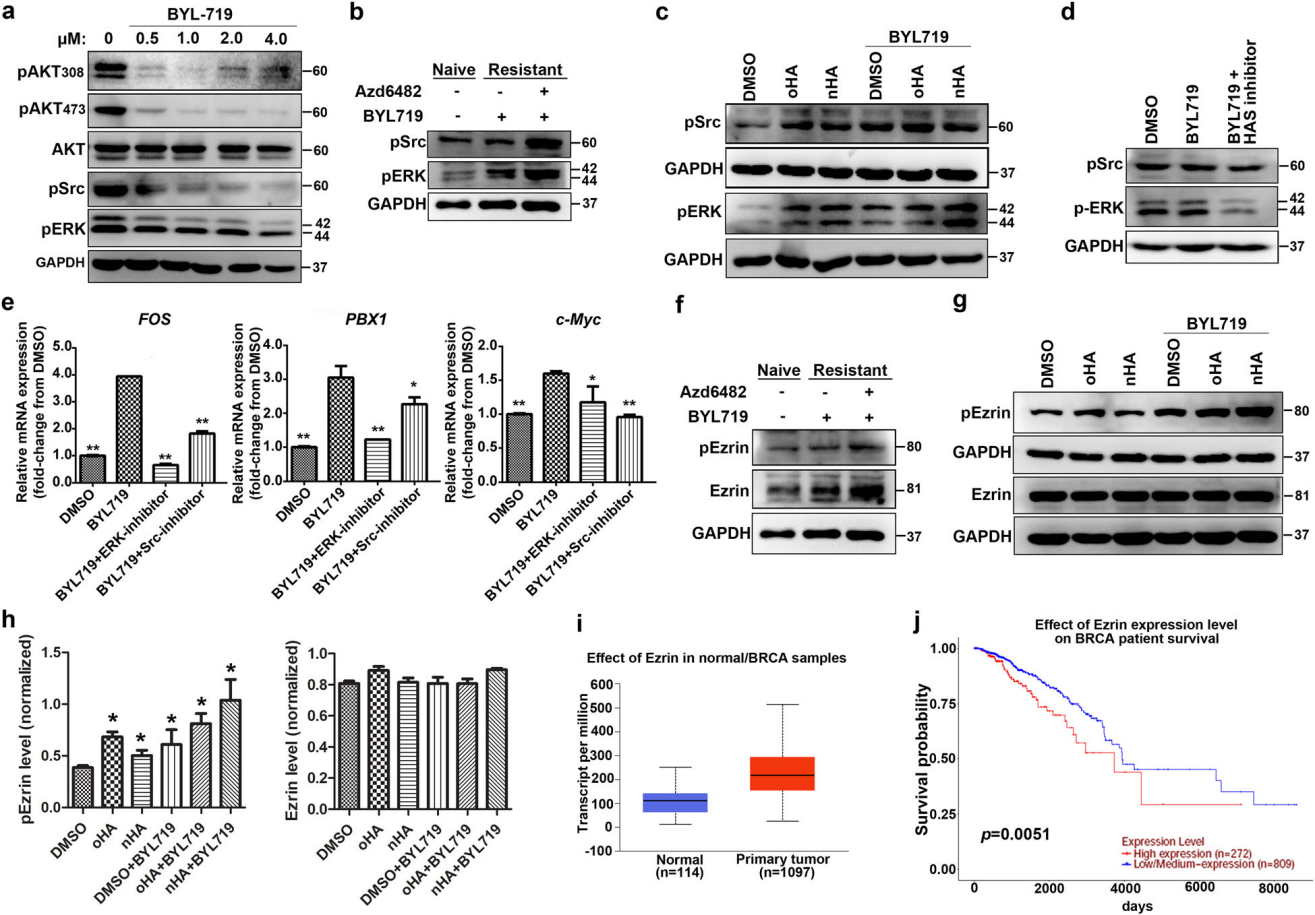
CD44/HA binding activates the Src-ERK-Ezrin signaling cascade. **a** The effects of BYL719 on PI3K/AKT/mTOR and Src-ERK signaling in luminal breast cancer cells were determined by immunoblotting. Cells were treated with BYL719 at different concentrations (0, 0.5, 1.0, 2.0, 4.0 μM). Western blot analyses and quantification were performed as described in the “Methods” section. **b** The activation of Src and ERK in sensitive and resistant MCF7 cells upon BYL719 (1.0 μM) treatment or combined treatment with BYL719 and the p110β inhibitor Azd6482 (1.0 μM) was determined by immunoblotting. **c** The effect of HA on the activation of Src and ERK in the presence of BYL719 was evaluated by immunoblotting. Exogenous oHA (20 μg/ml) or nHA (20 μg/ml) was added to stimulate MCF7 cells for 4 h to activate the CD44-HA signaling pathway in the presence or absence of BYL719. **d** An HAS inhibitor (4-methylumbelliferone, 0.2 μM) was used to disrupt the HA-CD44 interaction in MCF7/HAS2 cells, and its effects on the activation of Src and ERK were studied. **e** The expression of candidate target genes in MCF7 cells treated with DMSO, BYL719 (1 μM), BYL719 combined with a Src inhibitor (10 μM), or BYL719 combined with ERK inhibitor (25 μM) for 24 h were determined by RT-qPCR. Values are presented as the mean (±SD). All data represent at least three biological repeats. *p < 0.05, **P < 0.01, vs. BYL719 treatment group, *n* = 3. **f** The expression and activation of Ezrin in sensitive and resistant MCF7 cells upon BYL719 (1.0 μM) treatment or combined treatment with BYL719 and the p110β inhibitor Azd6482 (1.0 μM) were determined by immunoblotting. **g** The effect of HA on the activation of Ezrin in the presence of BYL719 was evaluated by immunoblotting. Exogenous oHA (20 μg/ml) or nHA (20 μg/ml) was added to stimulate MCF7 cells for 4 h to activate the CD44-HA signaling pathway in the presence or absence of BYL719. **h** Quantification of pEzrin and Ezrin signal from three biological repeats (statistical analysis: One way-ANOVA, *P < 0.05 vs. control). **i** Ezrin was upregulated in breast cancers from TCGA data. **j** Survival of breast cancer patients with high Ezrin protein levels (*Z* score >0) using TCGA data.

Given that the CD44-HA interaction mediates the coactivation of PI3K and Src/ERK signaling (4, 6, 8), we focused on alterations in their activation upon PI3Kα inhibition. BYL719 inhibited the activity of PI3K/AKT in a dose-dependent manner at early time points but partially attenuated the activation of Src and ERK (Fig. 7a). However, in BYL719-resistant cells, the phosphorylation level of Src/ERK was significantly increased and further enhanced when BYL719 treatment was combined with PI3Kβ inhibition (Fig. 7b). Moreover, HA stimulation enhanced the activation of Src and ERK in BYL719-sensitive cells, even when combined with BYL719 treatment (Fig. 7c). Disruption of the HA-CD44 interaction by an HAS inhibitor in MCF7/HAS2 cells attenuated the activation of ERK in the presence of BYL719 and had a weak inhibitory effect on Src activation (Fig. 7d). Therefore, the activation of the Src/ERK pathway (Fig. 7b and c) could contribute to resistance in BYL719-resistant cells. We then examined the effects of a Src inhibitor (SKI-606) and ERK inhibitor (U0126) on BYL719-induced ER-dependent transcription. Results showed that the treatment with U0126 or SKI-606 significantly reduced BYL719-activated transcription of the ER targets FOS, PBX1, and c-MYC (Fig. 7e), suggesting that the Src/ERK pathway is involved in modulating the ER activity.

As CD44 promotes breast cancer malignancy by interacting with cytoskeleton linker proteins, such as Ezrin, thus triggering the PI3K-related survival pathway^26–28^, we next determined the role of Ezrin in CD44/HA induced resistance. Indeed, in BYL719-resistant cells, the expression and phosphorylation levels of Ezrin were significantly upregulated and further enhanced with the addition of a PI3Kβ inhibitor (Fig. 7f). Moreover, exogenous HA slightly increased the activation of Ezrin in BYL719-sensitive cells, even when combined with BYL719 treatment (Fig. 7g and h). Additional analysis of TCGA database indicated that the increase of Ezrin in patients bearing either PIK3CA mutation or overexpression was closely associated with poor prognosis (Fig. 7i and j), further supporting that Ezrin contributes to resistance to BYL719. Collectively, the results suggested that CD44/HA signaling stimulate Src/ERK to activate Ezrin phosphorylation.

### Interconnected feedback loops among PIK3CA, HA/HAS2, ESRP1, and ER regulate CD44 alternative splicing and adaptive resistance

**Fig. 8:**
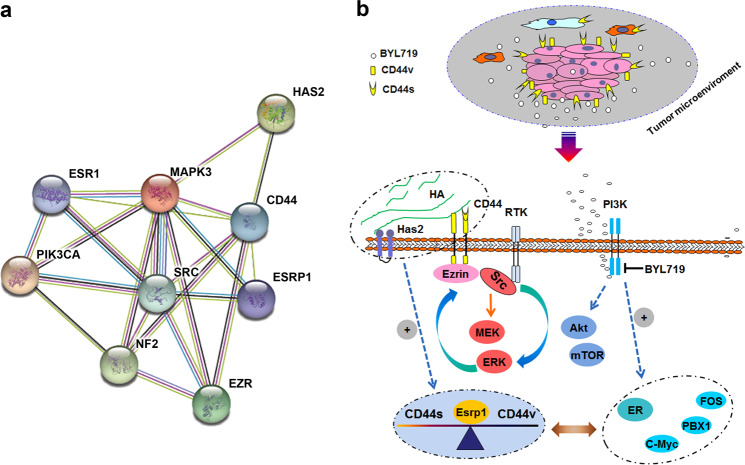
CD44 mediates resistance to PI3Kα inhibition through interconnected feedback loops consisting of CD44-ESRP1-Has2-ER. **a** Functional associations of the regulatory networks of CD44-correlated genes from analysis of STRING data are presented. **b** Scheme summarizing the proposed mechanism by which CD44 drives resistance to PI3Kα in luminal breast cancer. Upregulation of CD44 and the interaction of CD44 with HA leads to interconnected feedback loops consisting of CD44-ESRP1-Has2-ER, generating robust compensatory activation of the Src-ERK-Ezrin signaling cascade and dynamic regulation of the transition from a sensitive to resistant phenotype. The combination of PI3Kα inhibition with CD44 or HA suppression prevents this effect by blocking the Src/MAPK axis, resulting in superior antitumor activity.

Our results and analysis of data from TCGA revealed not only a positive relation between CD44 and HAS2, but also a parallel correlation between ER activity and CD44 aberrant splicing. Moreover, the analysis of the Search Tool for the Retrieval of Interacting Genes/Proteins (STRING) database showed tightly connected networks consisting of the PI3K and Src-ERK-Ezrin pathways, as well as ER transcription (Fig. 8a). Given that Src kinase could activate PI3K/AKT^19,20^ and ER signaling^21^, the activated signaling circuits investigated in our study might explain how CD44^high^ state acquired due to the development of BYL719-resistance leads to the reactivation of AKT/mTOR signaling in resistant cells (summarized in Fig. 8b). Collectively, the data suggested that interconnected feedback loops consisting of CD44-ESRP1-HA/Has2-ER occur in response to PI3Kα inhibition.

## Discussion

In this work, we show that luminal breast cancer cells escape the antitumor activity of PI3Kα inhibition via CD44 abnormal splicing and that the subsequent increase in the CD44-HA interaction initiates Src-ERK signaling cascades, which maintained AKT and mTOR activities in the presence of PI3Kα inhibitor. Evidence has shown that the therapeutic resistance is partially developed through the plasticity of cancer cell states. Recently, we reported a CD44^high^ state that acts an acquired response upon exposure to microenvironmental stimuli to promote malignancy in breast cancer^16^. This plasticity may be a shared feature of luminal BrCas that can generate adaptive resistance and tumor recurrence^29,30^. Therefore, we assume that the inducible acquisition of CD44 and its consequences account for the mechanism by which cancer cells reduce PI3Kα inhibition and maintain AKT/mTOR activation.

This work reveals that a CD44^high^ state due to enhanced alternative splicing was acquired upon PI3Kα inhibition in luminal BrCas, which mediates adaptive resistance to PI3Kα inhibitor. Coinhibition of PI3Kα and PI3Kβ further enhanced the aberrant splicing of CD44, suggesting a close relationship between adaptive PI3K-inhibitor resistance and the splicing of CD44. Interestingly, we found that ESRP1 was significantly upregulated during the development of resistance, which controls CD44 alternative splicing and leads to enhanced levels of CD44v and decreased levels of CD44s^11^. However, we found that the levels of CD44s and CD44v were simultaneously increased upon PI3K inhibition, implying that CD44 aberrant alternative splicing occurred in resistant cells. Previous studies have indicated that the alternative splicing of genes contributes to therapeutic resistance^31–33^. Similarly, we showed that the alternative splicing of CD44 was closely related to the adaptive response and plasticity of cancer cells.

Further, we analyzed signaling activation and identified a phenotype with hybrid E/M features in BYL719-resistant cells. As suggested previously, hybrid E/M cells showed increased tumor initiation and possessed increased plasticity^34,35^. Moreover, CD44s and CD44v are typically found in mesenchymal or epithelial cells, respectively^11^. Herein, our results that the simultaneously high levels of CD44s and CD44v may give rise to the hybrid E/M phenotype in BYL719-resistant cells.

Accumulating evidences have suggested that extensive crosstalk between PI3K and ER pathways^36^, as well as upregulation of ER-dependent transcription, could contribute to BYL719 resistance in PIK3CA-mutant BrCas^6,25^. In this study, a positive correlation between the expression of CD44 and ER was found in PIK3CA-mutant luminal BrCas from the TCGA data. In addition, others reported that ER activation could trigger p53-mediated repression in luminal-like BrCas^37,38^, and the inactivation of p53 could induce the derepression of CD44^39^. Consistently, our data indicated that the activation of ER with estradiol treatment drove an increase in CD44 splicing, while the ER degrader fulvestrant significantly reduced CD44 expression, providing direct evidence of a positive correlation between CD44 and ER activity. Moreover, we noted that CD44 was indispensable for ERα activation upon PI3Kα inhibition, suggesting that crosstalk between CD44 and ER activity occur in response to PI3K inhibitors.

It was reported that reactivation of PI3Kβ could compensate for PI3Kα inhibition^40^, and PDK1-SGK1 signaling could sustain mTORC1 activity in a PI3K-independent manner^41^. However, these mechanisms could not yet adequately elucidate the resistance to PI3Kα inhibitors. Here, we demonstrated that the CD44-mediated Src/ERK pathways can form a signal circuit that leads to resistance to PI3Kα inhibitors. As mentioned previously, CD44/HA are coactivators of the PI3K/AKT/mTOR and MAPK/ERK signal cascades^14^. Encouragingly, our STRING analysis also indicated a positive correlation between CD44-HAS2-Src/ERK/Ezrin signal cascades and PIK3CA signaling in aggressive BrCas. Further experiments showed that HAS2 not only enhanced the splicing of CD44 but also upregulated ESRP1. In addition, our luminal BrCas TCGA data analysis confirmed the positive correlation of CD44 and HAS2, which is in accordance with previous reports^42^. Based on these results, we conclude that sustained activation of AKT/mTOR in BYL719-resistant cells may result from increased CD44/HA signaling.

Taken together, we identified a feedback loop involving CD44 and ER activity and a positive correlation of CD44 and HAS2, which lead to BYL719-resistance. Subsequently, the enhanced activation of CD44-dependent Src/ERK signaling by ER or HAS2 further highlights the importance of these interconnected feedback loops in PIK3CA-mutant BrCas and their complex interactions that give rise to the highly dynamic regulation of transition from a BYL719-sensitive to BYL719-resistant phenotype.

## Conclusion

Our study revealed that CD44 alternative splicing occurred upon PI3K inhibition and CD44-HA signaling may activate a dynamic network of compensatory responses. Interconnected feedback loops consisting of CD44-ESRP1-Has2-ER may give rise to resistant phenotype. We also found a close association between ERα signaling and CD44 splicing. This study provides new insight into the mechanism of adaptive resistance to PI3Kα inhibition and suggests a supportive therapy by targeting CD44/HA in combination with PI3K inhibition.

## Material and methods

### Antibodies and reagents

Primary antibodies used were listed in Supplementary Table 3. Alpelisib (BYL719), Azd6482, and 17β-Estradiol (E2) were purchased from MCE (MedChemExpress). Src inhibitor (SKI-606) was obtained from Selleckchem (Houston, TX, USA). ERK inhibitor (U0126) was purchased from Cell Signaling Technology (Danvers, MA, USA).

### Generation of resistant cells

The MCF-7 and T47D cells were purchased from the American Type Culture Collection (ATCC) and cultured in MEM or DMEM, supplemented with 10% fetal calf serum (FBS). Resistant cells were generated by continuous treatment with BYL719 for 6 months at increasing concentrations^43^. Cells were maintained in phenol red-free medium with 1 µM of BYL719, supplemented with 5% charcoal/dextran-treated FBS.

### ER phosphomutants

Different phosphomimetic or phospho-dead ER constructs were generated as previously reported^44,45^. Full-length human ESR1 plasmid (NM_001291230) was obtained from Shanghai Jikai Biotechnology. ESR1 in which two codons encoding for serine (S) residues (S118 and S167) were mutated to encode either an aspartic acid (D) residue or glycine (G) residue were prepared. All mutations were verified by DNA sequencing.

### TCGA data analysis

Analyses of TCGA data from primary breast tumor samples (*n* = 1097) were performed with both RNA sequencing data and clinical annotations. Gene expression was log2 transformed. The clinical characteristics of each sample were downloaded directly from TCGA-BRAC. RNA sequencing data from a total of 543 luminal-like samples (ER^+^/Her2−) were obtained. Data from PIK3CA-associated luminal breast cancers were extracted from the TCGA database and then analyzed according to the presence or absence of mutant PIK3CA alleles and grouped as cases with mutant PIK3CA alleles (*n* = 230) or WT PIK3CA cases (*n* = 313).

### Statistical analyses

Statistical analyses were performed using GraphPad InStat software (GraphPad Software, Inc.). Nonparametric Mann–Whitney tests were performed to assess differences in CD44 expression. The significance of differences among groups was determined by one-way ANOVA, t-test, or Fisher exact test. Statistical significance was defined at *p* < 0.05.

## Supplementary information

Supplementary Material

Supplementary Figure 1

Supplementary Figure 2

Supplementary Figure 3

Supplementary Figure 4

Supplementary Figure 5
