# Supplementary material for: CD44/HA signaling mediates acquired resistance to a PI3Kα inhibitor
Source: Cell Death Dis. 2020 Oct 6;11(10):831. doi: 10.1038/s41419-020-03037-0 (PMC7538592; doi:10.1038/s41419-020-03037-0)
Supplement: Supplementary file 1 — Supplementary Material [file 41419_2020_3037_MOESM1_ESM.docx]

**Supplementary Material**

**Figure S1** **Effects of CD44 knockdown on ER activation and cell cycle upon PI3K pathway inhibition**

(a) Expression of phosphorylated ER (Ser104 and Ser106) in control or sh-CD44-transfected MCF7 cells was determined by immunoblotting. After depleted of hormones for 3 days, cells were subsequently treated with DMSO, BYL719 (1 μM), or combination BYL719 and Azd6482 (1 μM) treatment for 3 days.

(b) Effects of BYL719 and E2 treatment on cell cycle in MCF7 or sh-CD44 MCF7 cells. Cell cycle analysis of MCF7 or sh-CD44 MCF7 cells treated in hormone-depleted cells for 3 days and treatment with DMSO, E2 (100 nM), BYL719 (1 μM), or combined with BYL719 (1 μM) and E2(100 nM) for 24 h was performed using flow cytometry.

**Figure S2 Overexpressing phospho-dead and phosphomimetic mutants of phosphorylated ER (Ser118 and Ser167)**

Exogenously phosphomimetic and phospho-dead ER (S118) and ER (S167) were prepared. Two codons of ESR1 gene encoding for serine (S) residues (corresponding to S118 and S167) were mutated to encode either an aspartic acid (D) residue (ER118S→118D, ER167S→167D) or glycine (G) residue (ER118S→118G, ER167S→167G). Then wild-type and mutant ER ( phosphomimetic (S118D and S167D) and phospho-dead (S118G and S167G)) were expressed in naïve MCF7 or Sh-CD44 MCF7 cells by lentivirus transfection and determined by immunoblotting. An empty vector was used as a negative control (vector).

**Figure S3 HA completely attenuated the BYL719-induced inhibition to AKT signaling**

Western blot for pAKT(308) from MCF7 cells upon 24 hr of treatment with 10μg/ml oHA or 10μg/ml nHA with and without 1 μM BYL719.

**Figure S4 The effect of HA on BYL719 inhibition in CD44 knockdown cells**

The Akt/mTOR signaling was determined in MCF7/Sh-CD44 cells treated for 3 days with 1 μM BYL719 in the presence of 10μg/ml oHA or 10μg/ml HA as indicated.

**Figure S5 The expression of hyaluronan synthases (HAS2) in BYL719-resistant cells**

The expression of hyaluronan synthases (HAS2) was analyzed using cell lysates from BYL719-sensitive (S) and BYL719-resistant (R) MCF7 cells by western blot analysis.

**Supplemental Table 1 Correlation between CD44 expression and the clinico-pathological characteristics in the studied cohort**

| Tissue marker | ER+ | | | |  |  | ER- | |  | |  | |
| --- | --- | --- | --- | --- | --- | --- | --- | --- | --- | --- | --- | --- |
|  | CD44 expression | | | Signiﬁcance |  | CD44 expression | | | | | Signiﬁcance | |
|  | n | Median | 95% CI | *P* value |  | n | | Median | | 95% CI | | *P* value |
| Age |  | | |  |  |  | |  | |  | |  |
| <50 y | 51 1680 1987-3328 | | | 0.7901 |  | 43 | | 3520 | | 3236-12000 | | 0.2559 |
| ≥50 y | 78 1997 2073-3028 | | |  |  | 69 | | 5360 | | 7052-15388 | |  |
| Tumor size |  | | |  |  |  | |  | |  | |  |
| <4 cm | 28 2244 1869-3658 | | | 0.6478 |  | 35 | | 3700 | | 4437-19646 | | 0.3355 |
| ≥4 cm | 101 1818 2111-2980 | | |  |  | 77 | | 4162 | | 5933-11737 | |  |
| Histologic grade |  | | |  |  |  | |  | |  | |  |
| 1-2 | 116 2022 2265-3096 | | | 0.2736 |  | 75 | | 3696 | | 5270-12310 | | 0.3340 |
| 3 | 15 1522 1065-2965 | | |  |  | 37 | | 5620 | | 5932-17987 | |  |
| Lymphnode metastasis |  | | |  |  |  | |  | |  | |  |
| Yes | 28 1716 1579-2860 | | | 0.3166 |  | 25 | | 3520 | | 3854-14274 | | 0.7887 |
| No | 101 | 1996 | 2233-3160 |  |  | 87 | | 4162 | | 6390-13728 | |  |

**Supplemental Table 2 List of primers used in the study**

| Primer |  | Sequence: 5’-3’ |
| --- | --- | --- |
| ESRP1 | Forward | CAATATTGCCAAGGGAGGTG |
|  | Reverse | GTCCCCATGTGATGTTTGTG |
| C-myc | Forward | CCTCCACTCGGAAGGACTATC |
|  | Reverse | TGTTCGCCTCTTGACATTCTC |
| PBX1 | Forward | CAGTGAGGAAGCCAAAGAGG |
|  | Reverse | CAGCTGTTTTGGCAGCATAA |
| FOS | Forward | GACTGATACACTCCAAGCGG |
|  | Reverse | CATCAGGGATCTTGCAGGC |
| CD44-pattern | Forward | AGTCACAGACCTGCCCAATGCCTTT |
|  | Reverse | AGTCACAGACCTGCCCAATGCCTTT |
| GAPDH | Forward | AACGGATTTGGTCGTATTGGG |
|  | Reverse | TCGCTCCTGGAAGATGGTGAT |

**Supplemental Table 3 List of primary antibodies, source and dilution information of different primary antibodies used in the study**

| **Protein** | **Catalog No.** | **Clone(if monoclonal)** | **Manufacturer** | **Application** | **Dilution** |
| --- | --- | --- | --- | --- | --- |
| pAKT308 | 13038 | N/A | cell signaling technology | Western blot | 1:1000 |
| pAKT474 | 4060 | N/A | cell signaling technology | Western blot | 1:2000 |
| AKT | 4691 | N/A | cell signaling technology | Western blot | 1:1000 |
| p-mTOR | 5536 | N/A | cell signaling technology | Western blot | 1:1000 |
| mTOR | 2983 | N/A | cell signaling technology | Western blot | 1:1000 |
| GAPDH | Mab-5465-100 | N/A | Multi Sciences | Western blot | 1:1000 |
| Zeb1 | 3396 | N/A | cell signaling technology | Western blot | 1:1000 |
| Slug | 9585 | N/A | cell signaling technology | Western blot | 1:1000 |
| β-Catenin | ab32572 | N/A | Abcam | Western blot | 1:1000 |
| Sox2 | 3579 | N/A | cell signaling technology | Western blot | 1:1000 |
| Lamin | ab133741 | N/A | Abcam | Western blot | 1:1000 |
| CD44 | Ab189524 | N/A | Abcam | Western blot | 1:1000 |
| CD44 | BE0039 | IM7 | BioXcell | Blocking | - |
| Normal IgG | BE0097 | IgG2a Fc | BioXcell | Blocking | - |
| ESRP1 | ab107278 | N/A | Abcam | Western blot | 1:1000 |
| ERα | ab32063 | N/A | Abcam | Western blot | 1:1000 |
| pER118 | Ab32396 | N/A | Abcam | Western blot | 1:1000 |
| pER167 | 64508 | N/A | cell signaling technology | Western blot | 1:1000 |
| S6 | 2217 | N/A | cell signaling technology | Western blot | 1:1000 |
| p-S6 235/236 | 4858 | N/A | cell signaling technology | Western blot | 1:1000 |
| p-S6 240/244 | 5364 | N/A | cell signaling technology | Western blot | 1:1000 |
| pSrc | 6943 | N/A | cell signaling technology | Western blot | 1:1000 |
| pERK | 4370 | N/A | cell signaling technology | Western blot | 1:1000 |
| Ezrin | 3145 | N/A | cell signaling technology | Western blot | 1:1000 |
| pEzrin | 3726 | N/A | cell signaling technology | Western blot | 1:1000 |
| HAS2 | Ab131364 | N/A | Abcam | Western blot | 1:1000 |
| pER104 | AF3059 | N/A | Affinity | Western blot | 1:1000 |
| pER106 | AF3060 | N/A | Affinity | Western blot | 1:1000 |

**Supplemental Experimental Procedures**

**1. shRNA-mediated gene knockdown**

Cell lines stably expressing Has2 were prepared through lentiviral transduction as previously described [^1^](#_ENREF_1). The pCMVIE-IRES-HAS2 vector encoding HAS2 was purchased from Hanbio (Shanghai, China). Stable Has2-expressing cells were chosen using puromycin. Human CD44 shRNA (pLKD-CMV-G&PR-U6-shRNA/CD44), which targets all CD44 isoforms, and control GFP shRNA were obtained from Obio Technology (Shanghai) Corp., Ltd. Stably transfected cells were purified by FACS with a GFP marker.

**2. Mice**

MMTV-PyMT (FVB/n) and SCID mice were acquired from the Model Animal Research Center of Nanjing University. All protocols involving mice were evaluated and approved by our Institutional Animal Care and Use Committee and performed under veterinary supervision. To explore resistance to BYL719, the MMTV-PyMT mouse was used. Mice were monitored weekly for mammary tumor development by palpation until one of the tumors in each mouse reached an approximately average size of 250-400 mm^3^. Then mice were randomized into control and treatment groups (n = 6 per group). Mice were treated daily with BYL719 by oral gavage (25 mg/kg) for 21 days, which was dissolved in 0.5% carboxymethyl cellulose sodium salt (Sigma). Tumor size was measured twice a week using a caliper.

**3. Cell survival analysis** The survival of cancer cells lines upon BYL719 treatment was determined by CCK-8 assay (Dojindo, Japan). Approximately 3.5×10^3^ purified cells in 100 ml were incubated in five duplicate wells in 96-well plates. After BYL719 exposure for different durations, CCK-8 reagent (10 ml) was added to each well and incubated at 37°C for 2 h. The optical density at 450 nm was measured using an automatic microplate reader (Synergy 4; BioTek, Winooski, VT, USA).

**4. Analysis of CD44 expression patterns by RT-PCR** Cells were harvested, and total RNA was purified using TRIzol reagent. For reverse transcription, random primers were used. To determine CD44 expression patterns, we used a human-specific primer pair spanning the entire variant region (Supplementary Table 2). All CD44 variants were theoretically amplified. The PCR mixture was obtained from Takara.

**5. Cell cytotoxicity in a 3D culture system**

We purified either CD44^high^ or CD44^low^ subpopulations of MMTV-PyMT tumor cells by FACS and cultured them in 96-well ultralow attachment plates for 2 days to form tumor spheroids. Then, the tumor spheroids were placed on collagen/Matrigel 3D matrix with or without BYL719 (1 μM). Microscopic imaging was performed every day to monitor tumor growth. After 3 days, apoptotic cells were detected using an in situ cell death detection kit (LIVE/DEAD Cell Imaging Kit (488/570)) (Invitrogen, Carlsbad, CA, USA). The live and dead cells were observed with the green (ex/em, 488 nm/515 nm) or red (ex/em 570, nm/602 nm) fluorescence channel, respectively.

**6. Immunohistochemical staining**

The expression of CD44 was determined by immunohistochemical staining. Paraffin-embedded human BrCa sections of ER^+^ (n=129) or ER^-^ (n=112) tumor samples were obtained from US Biomax (BR1504, BR1505) and Shanghai Superchip Biotech (HBre-Duc052Bch, HBre-Duc068Bch-01, OD-CT-RpBre03-004). Informed consent was obtained from all subjects. Paraffin-embedded sections were fixed in acetone. After elimination of endogenous peroxidase activity (0.3% H_2_O_2_, 20 min), tissues were rehydrated with PBS. Then, sections were treated with primary anti-CD44 antibody for 60 min at room temperature. Detection was achieved with an appropriate secondary antibody (Invitrogen, CA, USA) and a HRP-conjugated ABC amplification system.

**7. Quantitative real-time PCR**

Real-time PCR was performed to determine the expression of ER-dependent transcription factors PBX1, cFOS, and c-MYC. Total RNA was extracted using an RNeasy Mini kit (Takara, Dalian, China). For real-time PCR quantitation, reverse transcribed cDNAs were prepared with a First-Strand cDNA Synthesis Kit (Takara, Dalian, China) and amplified with the primers (Supplementary Table 2) and SYBR Green mix using the ABI 7500 system (Applied Biosystems). Relative RNA expression of each specific gene over the housekeeping gene GAPDH was analyzed by the change-in-threshold (2^-∆∆CT^) method.

1. Zhang, G. *et al*. A novel role of breast cancer-derived hyaluronan on inducement of M2-like tumor-associated macrophages formation. *Oncoimmunology.* **5,** e1172154 (2016).
