# Supplementary figures and images for: CD44/HA signaling mediates acquired resistance to a PI3Kα inhibitor
Source: Cell Death Dis. 2020 Oct 6;11(10):831. doi: 10.1038/s41419-020-03037-0 (PMC7538592; doi:10.1038/s41419-020-03037-0)

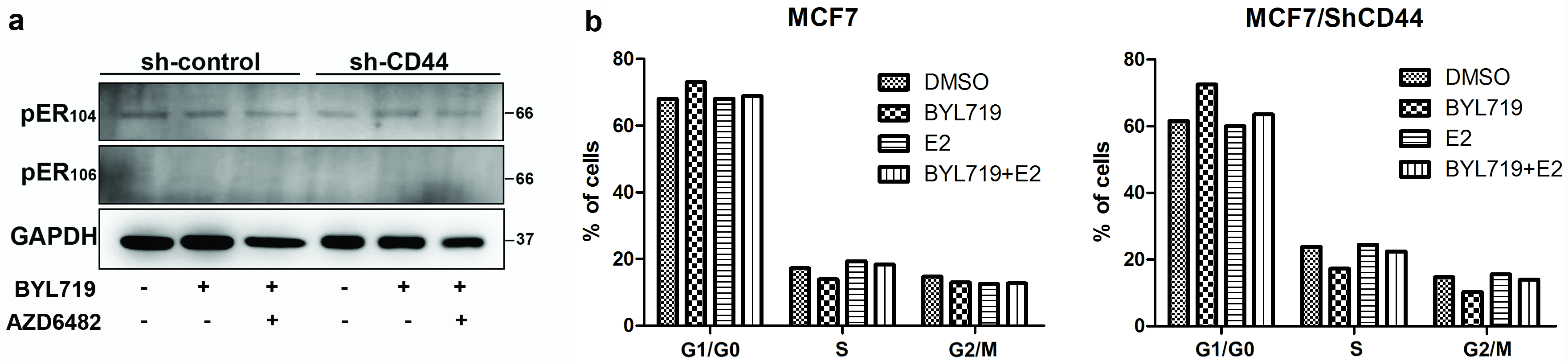

Supplement: Supplementary file 2 — Supplementary Figure 1 [file 41419_2020_3037_MOESM2_ESM.tif]

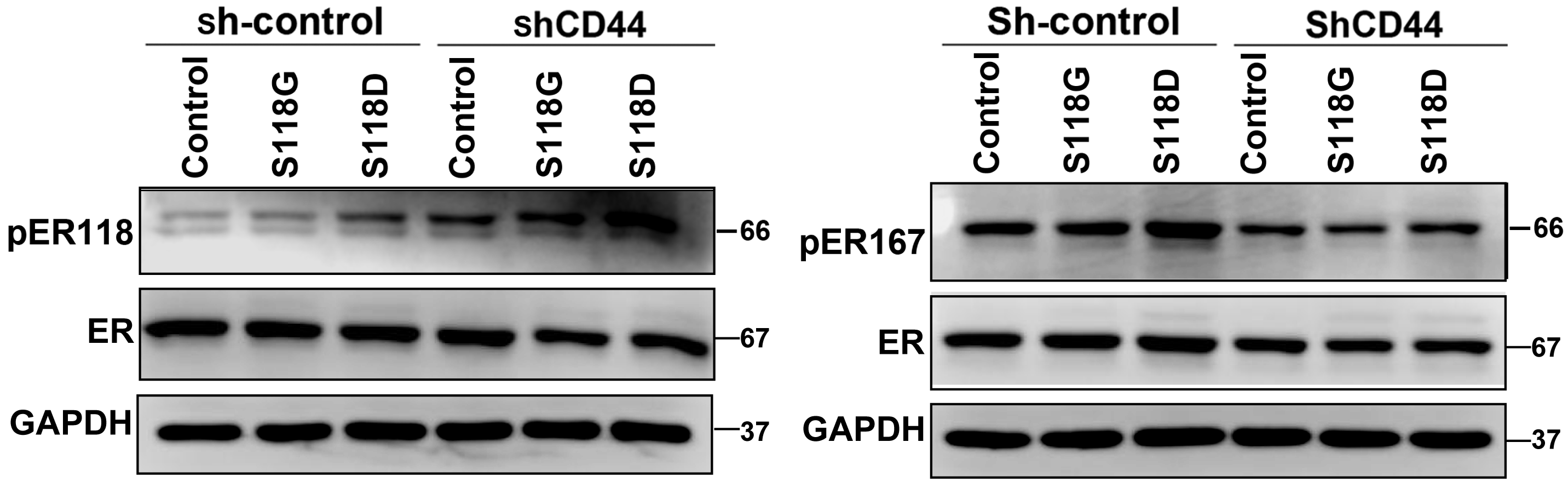

Supplement: Supplementary file 3 — Supplementary Figure 2 [file 41419_2020_3037_MOESM3_ESM.tif]

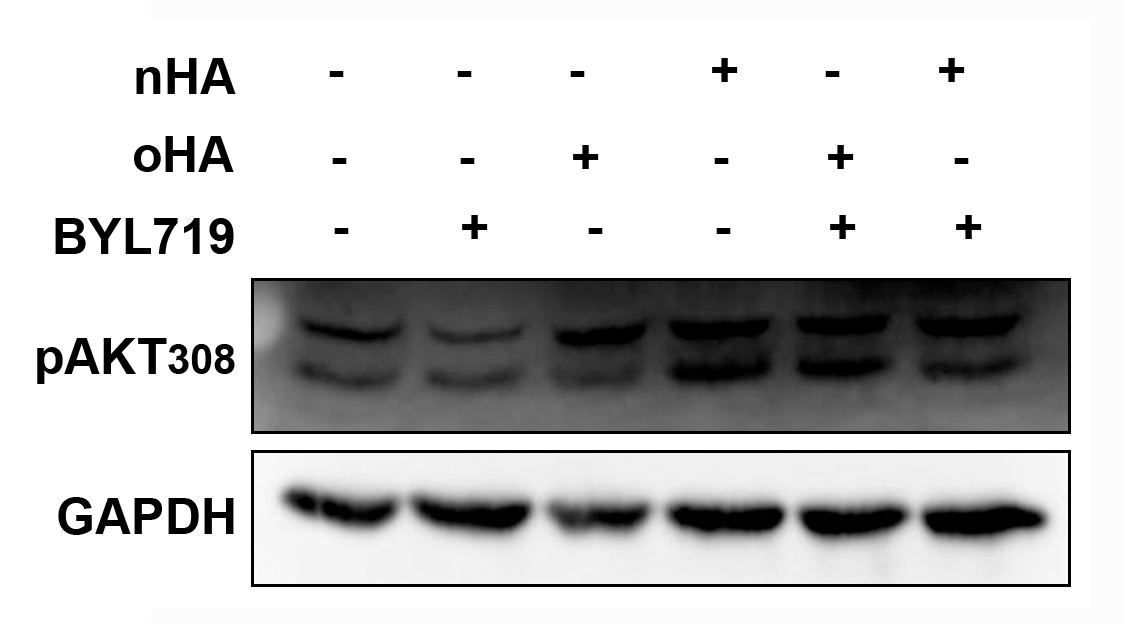

Supplement: Supplementary file 4 — Supplementary Figure 3 [file 41419_2020_3037_MOESM4_ESM.tif]

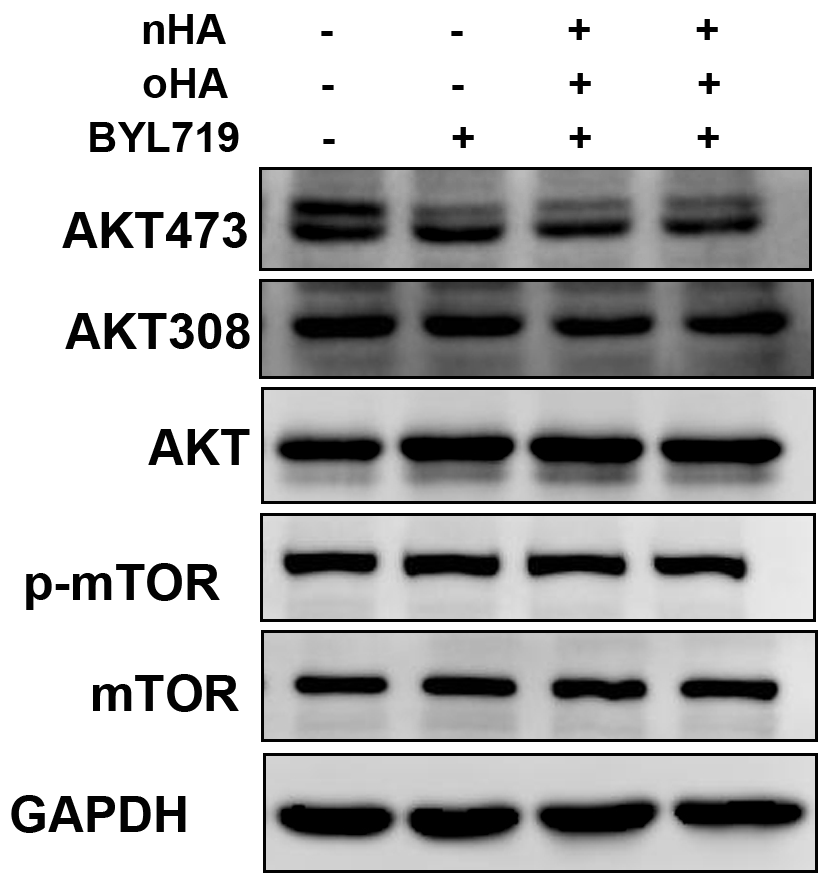

Supplement: Supplementary file 5 — Supplementary Figure 4 [file 41419_2020_3037_MOESM5_ESM.tif]

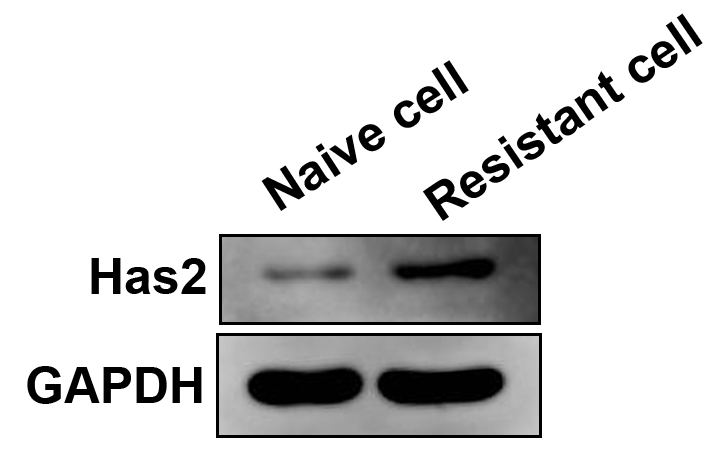

Supplement: Supplementary file 6 — Supplementary Figure 5 [file 41419_2020_3037_MOESM6_ESM.tif]
